# COVID-19: Mental Health Prevention and Care for Healthcare Professionals

**DOI:** 10.3389/fpsyt.2021.566740

**Published:** 2021-03-23

**Authors:** Julie Rolling, Amaury C. Mengin, Cédric Palacio, Dominique Mastelli, Morgane Fath, Adrien Gras, Jean-Jacques Von Hunolstein, Carmen M. Schröder, Pierre Vidailhet

**Affiliations:** ^1^Regional Center for Psychotraumatism Great East, Strasbourg University Hospital, Strasbourg, France; ^2^Medico-Psychological Emergency Unit, Strasbourg University Hospital, Strasbourg, France; ^3^Department of Psychiatry, Mental Health and Addictology, Strasbourg University Hospital, Strasbourg, France; ^4^CNRS UPR 3212, Institute for Cellular and Integrative Neurosciences, Strasbourg, France; ^5^INSERM U1114, Cognitive Neuropsychology, and Pathophysiology of Schizophrenia, Strasbourg, France; ^6^Cardiology Department, Strasbourg University Hospital, Strasbourg, France; ^7^Federation of Translational Medicine of Strasbourg, Strasbourg, France

**Keywords:** COVID-19, healthcare professionals, continuous stress exposure, mental health, psychological crisis prevention, pandemic

## Abstract

The Coronavirus Disease 2019 (COVID-19) pandemic exposed health professionals to high stress levels inducing significant psychological impact. Our region, *Grand Est*, was the most impacted French region during the first COVID-19 wave. In this context, we created CoviPsyHUS, local mental health prevention and care system dedicated explicitly to healthcare workers affected by the COVID-19 pandemic in one of this region's tertiary hospitals. We deployed CoviPsyHUS gradually in 1 month. To date, CoviPsyHUS comprises 60 mental health professionals dedicated to 4 complementary components: (i) a mental health support hotline (170 calls), (ii) relaxation rooms (used by 2,120 healthcare workers with 110 therapeutic workshops offered), (iii) mobile teams (1,200 contacts with healthcare staff), and (iv) a section dedicated to patients and their families. Among the critical points to integrate mental health care system during a crisis, we identified: (i) massive dissemination of mental health support information with multimodal communication, (ii) clear identification of the mental health support system, (iii) proactive mobile teams to identify healthcare professionals in difficulty, (iv) concrete measures to relieve the healthcare professionals under pressure (e.g., the relay in communication with families), (v) support for primary needs (body care (physiotherapy), advice and first-line therapy for sleep disorders), and (vi) psychoeducation and emotion management techniques. The different components of CoviPsyHUS are vital elements in meeting the needs of caregivers in situations of continuous stress. The organization of 4 targeted, modular, and rapidly deployable components makes CoviPsyHUS an innovative, reactive, and replicable mental health prevention and care system that could serve as a universal support model for other COVID-19 affected teams or other exceptional health crises in the future.

## Introduction

**Figure 1:**
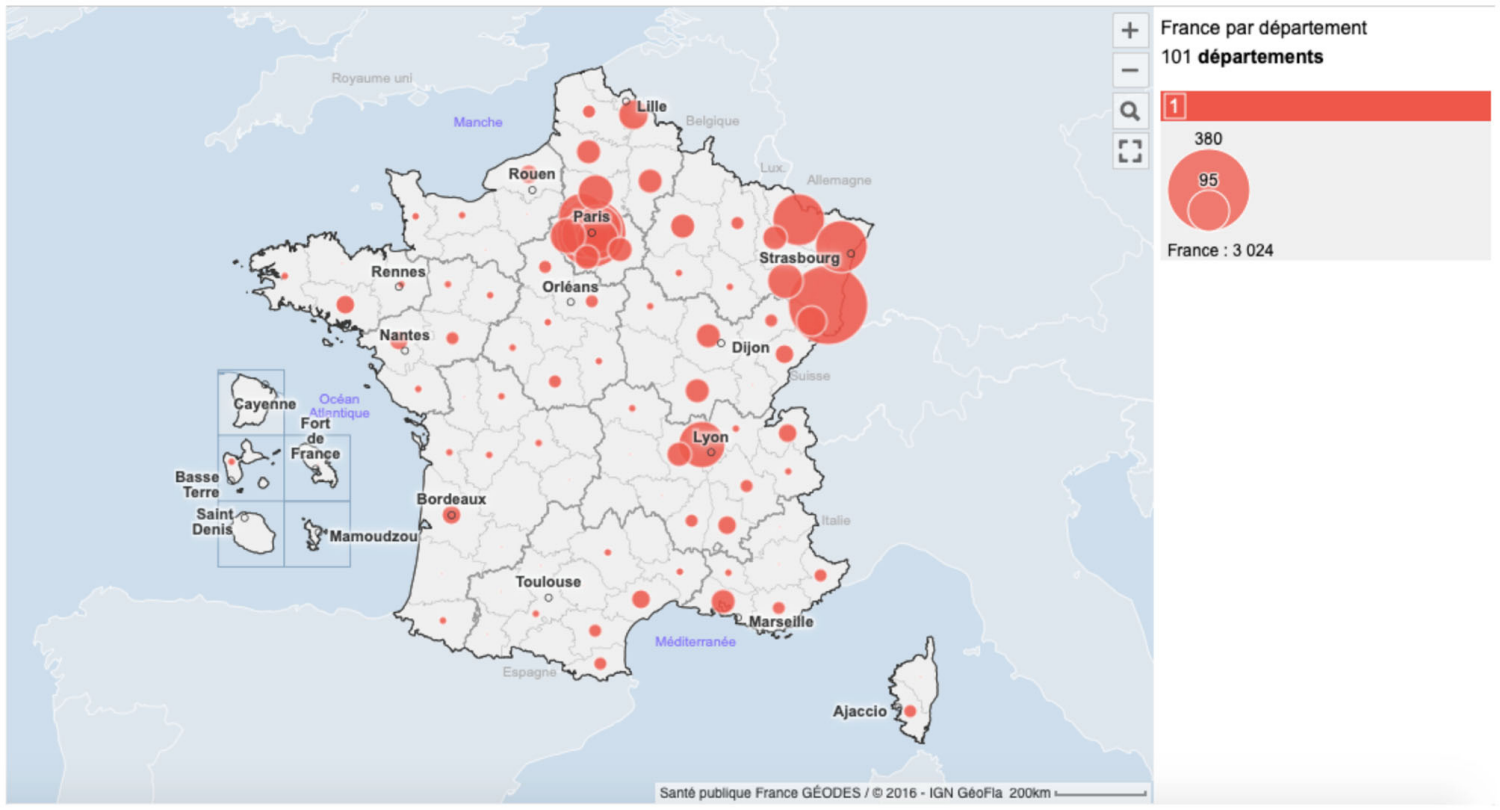
Cumulative number of people who died of COVID-19 in France as of March 30, 2020. *Santé Publique France Géodes*. The circles correspond to the mortality rate per 100,000 inhabitants. The largest circle corresponds to a mortality rate of 380 deaths per 100,000 inhabitants, and the smallest circle corresponds to a mortality rate of 95 deaths per 100,000 inhabitants.

In May 2020, the Coronavirus Disease 2019 (COVID-19) pandemic affected the entire world (1). In France, the *Grand Est* (region of eastern France) was early-on massively impacted by the COVID-19 (see Figure 1). This region was one of the two main initial disease clusters. Due to a gathering of 2000 people (February 24–29, 2020), a favorable context to intense interindividual contamination and viral spread throughout the *Grand Est* territory emerged. In total, to date (May 07, 2020), 12,274 people were hospitalized in the Great East, and 4,665 people have died, which corresponds to 237 hospitalizations and 89 deaths per 100,000 inhabitants.

In this context, healthcare practitioners of one of the *Grand Est* reference hospitals located in Strasbourg (*Hôpitaux Universitaires de Strasbourg*, HUS; Strasbourg University Hospital; 13,000 agents) worked hard in the care of COVID-19 patients. The sudden and massive arrival of hundreds of COVID-19 patients with severe symptoms required an unprecedented increase in intensive care beds' capacity with a transition from a usual capacity of 95 beds to 207 to cope with the patient influx. The health situation quickly required the transfer of patients under mechanical ventilation by train or military planes to other French hospitals and abroad. Like many frontline clinicians fighting COVID-19, they were subjected to high stress levels inducing significant psychological impact (2–7).

**Figure 2:**
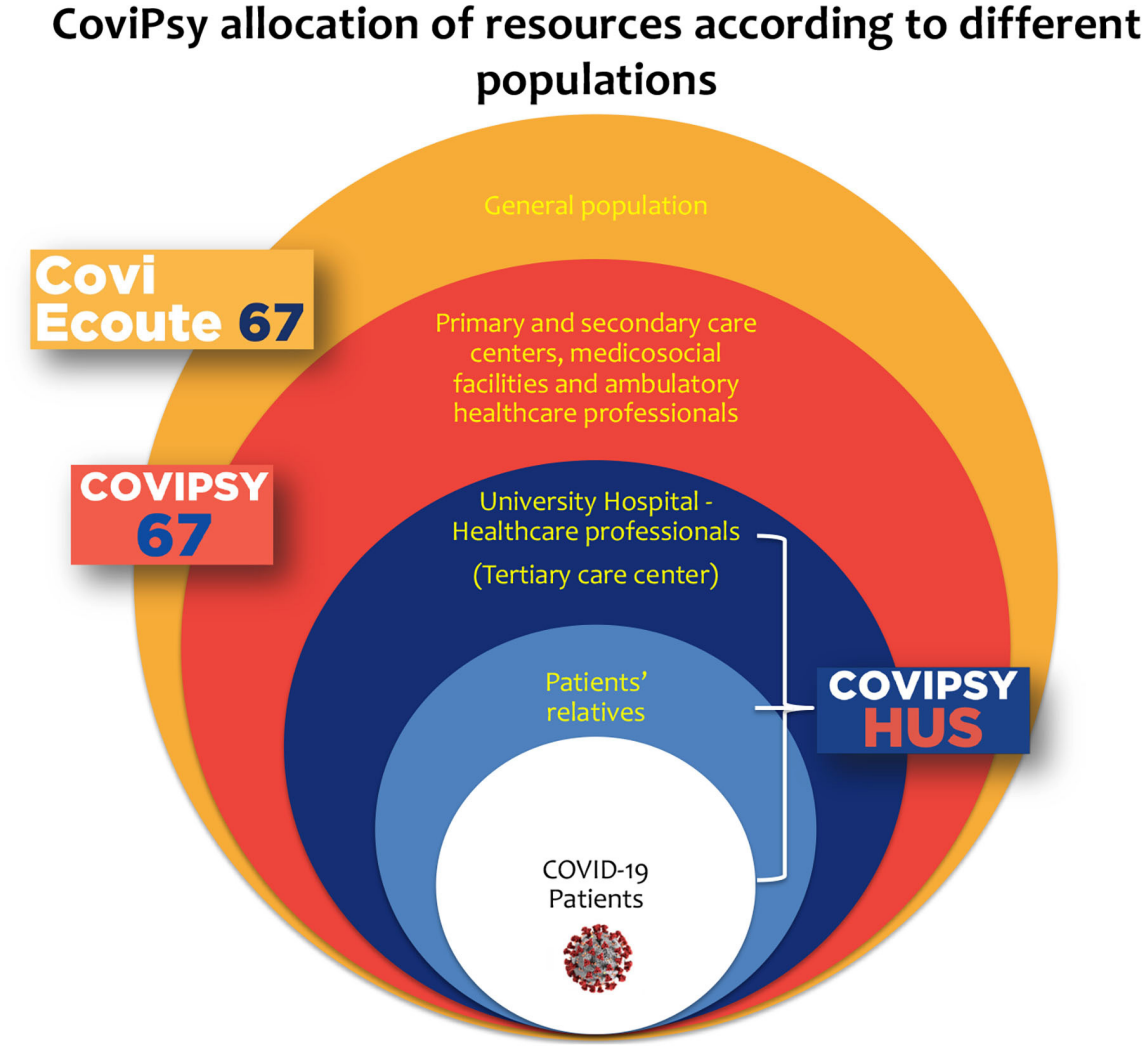
CoviPsy organization of resources allocation according to different populations.

Based on local, national, and international observations (8–10), the increasing need for mental health support for hospitalized patients and healthcare professionals became obvious. In this context, we created CoviPsyHUS, local mental health prevention and care system dedicated explicitly to healthcare workers affected by the COVID-19 pandemic in Strasbourg University Hospital. Initially inspired by our Asian colleagues' contributions, this system evolved to integrate caregivers' local needs optimally. We integrated CoviPsyHUS into a more extensive regional system (CoviPsy) organized concentrically according to the entire population's mental health needs, from the most exposed to the least exposed (see Figure 2). Indeed, previous studies highlighted the importance of such nested systems capable of guaranteeing essential care levels for all people in crisis times (11).

This article will focus on the innovative implementation of CoviPsyHUS - the subsection of CoviPsy dedicated to our hospital center - organization, clinical feedback, and progressive development. We will analyze critical elements of its rapidly deployable and efficient organization to transpose this innovative delivery of prevention and care in mental health to other teams and countries affected by COVID-19 or similar future exceptional health crises.

## Local Context

The care of COVID-19 patients required a rapid and massive daily reorganization of Strasbourg University Hospital activity. There was a need for changes in practices with new skills to be acquired quickly, new teams to integrate, and profound changes in relations with patients and families for all teams. In this context, Strasbourg University Hospital healthcare workers described a significant state of distress, mainly related to a feeling of vulnerability. Among distress factors, healthcare workers evoked: unfamiliarity with the virus, infectious risk (590 agents out of a total of 13,000 presented with COVID-19 symptoms), high morbidity and mortality, perceived insufficiencies of protective equipment, and unpredictability (patients' admission rapid increase, daily protocol changes). All these elements led to a feeling of losing control in a context where many professional (changes in attributed units, isolation) and personal standards (risk of contamination of loved ones, the anxiety of death, guilt, confinement) had changed. This exceptional health situation has led to unprecedented, regularly and long-lasting physical and mental pressure, placing great demands on caregivers' adaptive mechanisms to stress, both individually and collectively.

## Covipsy, A Primary Prevention and Mental Health Care System

In Strasbourg, the CoviPsy team's knowledge and skills in medical and psychological support during exceptional sanitary situations are based on both the local Medico-Psychological Emergency Unit (*Cellule d'Urgence Médico-Psychologique*, CUMP) and of the Regional Psychotrauma Center (*Centre Régional du Psychotraumatisme CRP Grand Est*). The CUMP (12) constitutes a unique French mental health emergency system for immediate on-site intervention during and after traumatic events. The French government has recently created ten regional CRP to enhance the expertise and interventions in managing psychotrauma in the long term and complicated situations such as those encountered with the COVID-19 pandemic. Both previously existing care systems thus combine clinical expertise, responsiveness, and organizational skills. The CoviPsy system benefited from and included professional teams from CUMP and CRP.

## Covipsyhus, A Primary Prevention and Mental Health Care System for COVID-19 Patients, Their Families, and Healthcare Professionals

**Figure 3:**
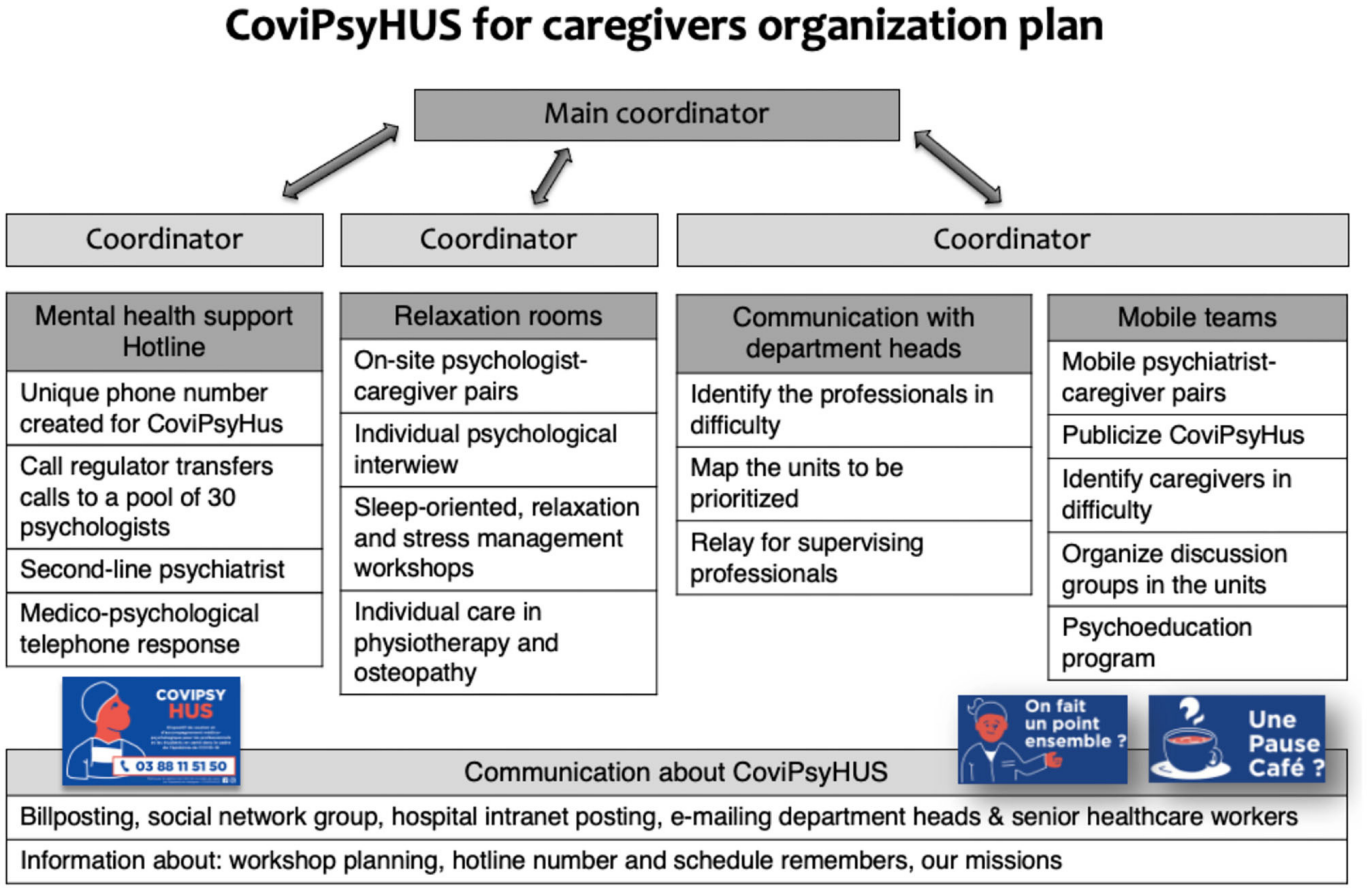
CoviPsyHUS organizational plan dedicated to healthcare professionals.

CoviPsyHUS involved mental healthcare professionals (adult psychiatrists, child and adolescent psychiatrists, psychologists, psychiatry nurses) allocated to different action sectors. This variety of actions offered a gradual preventive and care response based on the team's clinical needs (see Figure 3). The system's components' deployment was progressive, starting from the patients and families component to components dedicated to healthcare professionals to provide global mental health support.

### CoviPsyHUS Dedicated to Patients and Their Families

As the consultation-liaison psychiatry team usually consults in all University Hospital wards, they continued visiting patients who required psychiatric attention, although consultations by phone or through videoconference were preferred. However, the “COVID-19 patients and their families” component was a specific add-on to usual activities.

Indeed, we quickly identified families as particularly vulnerable due to highly restricted or prohibited visits and limited discussions with caregivers due to hospital staff's particular time constraints. Currently, available data are showing strong reactions of fear and panic and feelings of uncertainty among families (13), leading us to initiate a dedicated telephone relay with a double-entry system:

- a mental health support platform directly accessible to families,
- a direct telephone line for doctors in COVID-19 units to report families in need of specific support.

Intensive care clinicians mostly reported problematic situations concerning hospitalized patients or bereaved families.

Families warmly welcomed psychiatrists' supportive phone calls. This system branch was very beneficial to medical teams since it enabled them to feel supported in administrating care whenever they felt guilty for not having enough time to talk to families and address mental health issues. At the same time, they did their best to maintain contact with patients' families.

### CoviPsyHUS Dedicated to Healthcare Professionals

#### Mental Health Support Hotline

The mental health support hotline allowed hospital staff to get in touch, anonymously if desired, with psychologists and psychiatrists providing live teleconsultations, thus identifying vulnerable caregivers and organizing follow-up. The response was graduated with the possibility of face-to-face consultations and referral to a psychiatrist if necessary (5% of cases, for drug prescription or need for medical leave from work).

Though we largely publicized the hotline, hospital workers remained reluctant to call, and the hotline was underused, as previously in China (14, 15). Between March 23 and May 7, 2020, the hotline received 170 (70% of women and 65% of frontline caregivers) calls for 13,000 agents. Despite the underuse of the hotline in our system, its existence was fundamental. It must be maintained in similar organizations as it allowed many hospital workers to identify CoviPsyHUS (16). Indeed, all calls were justified and commonly required specialized follow-up. In this context, accessibility over an extended hourly period (9 A.M.−10 P.M.) favored its use by isolated hospital staff (e.g., for those on temporary leave due to COVID-19).

We hypothesize that this underuse was the consequence of several issues: the reluctance of healthcare professionals to spontaneously call for personal mental suffering during such a sanitary crisis; healthcare professionals identified the telephone system as an additional measure of distancing in a global situation of confinement. In this context, it was necessary to identify a physical place of care in the hospital.

#### Relaxation Rooms

**Figure 4:**
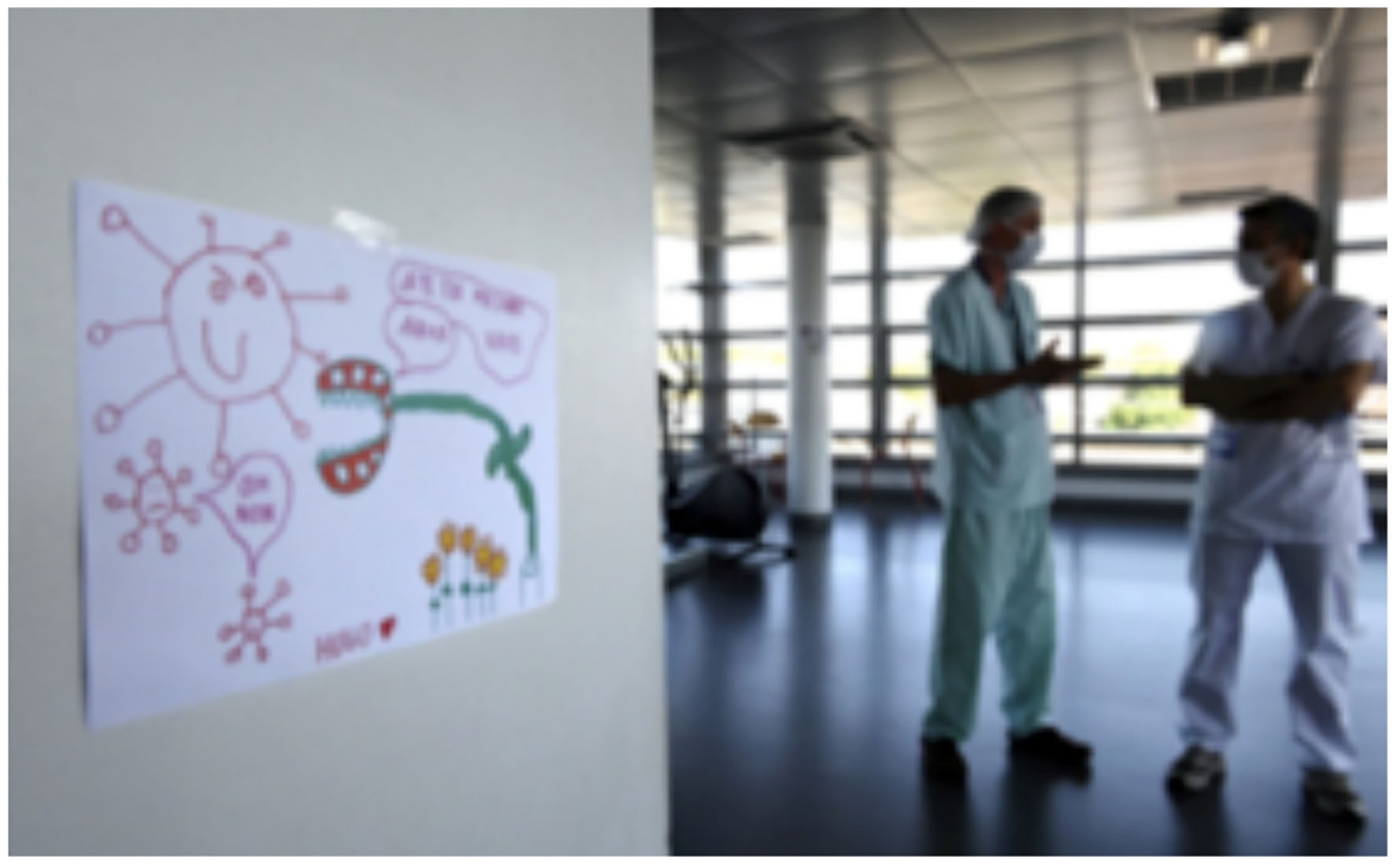
Psychological interview in a relaxation room.

Relying on our Chinese colleagues' experience who reported the importance for healthcare professionals to have a place to rest, we quickly opened relaxation rooms for hospital staff. Although a risk of contagion existed in a relaxation room, we privileged direct social interaction but applied specific protective measures (15) (see Figure 4). We set up these rooms to create different spaces (reception, individual and collective space). In these rooms, caregivers had the opportunity to take a break (CoviPsyHUS team members offered coffee and cookies to promote conviviality) out of their daily stressful clinical context. A psychologist and mental health caregivers were present every day to welcome hospital professionals and offer individual consultations. The CoviPsyHUS team provided several workshops: mind-body techniques (mindfulness, yoga, sophrology, hypnosis), body-centered techniques (osteopathy and physiotherapy), and sleep workshops. In total, to date, 110 workshops were led by 30 different professionals and volunteers.

In particular, the sleep workshops were essential. They addressed one of the significant acute stress symptoms reported by most healthcare workers: sleep disorders, notably insomnia and nightmares (17), thus preventing a vicious circle of psychological stress and insomnia (18). Indeed, some staff described an increase in substance intake in the evening, notably alcohol, for anxiolytic and hypnotic purposes. Even though sleep deprivation persisted throughout, the state of exhaustion only appeared later-on, which illustrates the importance of activated stress mechanisms maintaining high vigilance levels over an extended period.

Healthcare professionals frequently presented with emotional and mood dysregulation: irritability, anger, hypomania, depressive mood. Moreover, they often reported hyperarousal and peri-traumatic dissociation: time perception changes, autopilot mode functioning (“We were like robots”). These elements might predict the manifestation of Posttraumatic Stress Disorder (PTSD) in the long run. We know from the literature that caregivers are more at risk of developing PTSD than the general population (3, 19). Studies following exposure to SARS in caregivers show that infected caregivers have more psychic complications than other patients (6). So being a caregiver is associated with a risk factor of more severe and prolonged post-traumatic symptoms (6). However, as the COVID-19 outbreak consists of a long-lasting, ongoing and stressful event with an uncertain outcome, we consider that specific and rapid intervention in the heart of the critical event, mainly targeted on stress, sleep, and emotion regulation, is valuable to prevent future psychological disorders (19).

Body-centered therapies addressed a central need of hospital workers by removing muscular tensions. These workshops made it possible to take charge of the first manifestations of stress, which were very physical.

**Figure 5:**
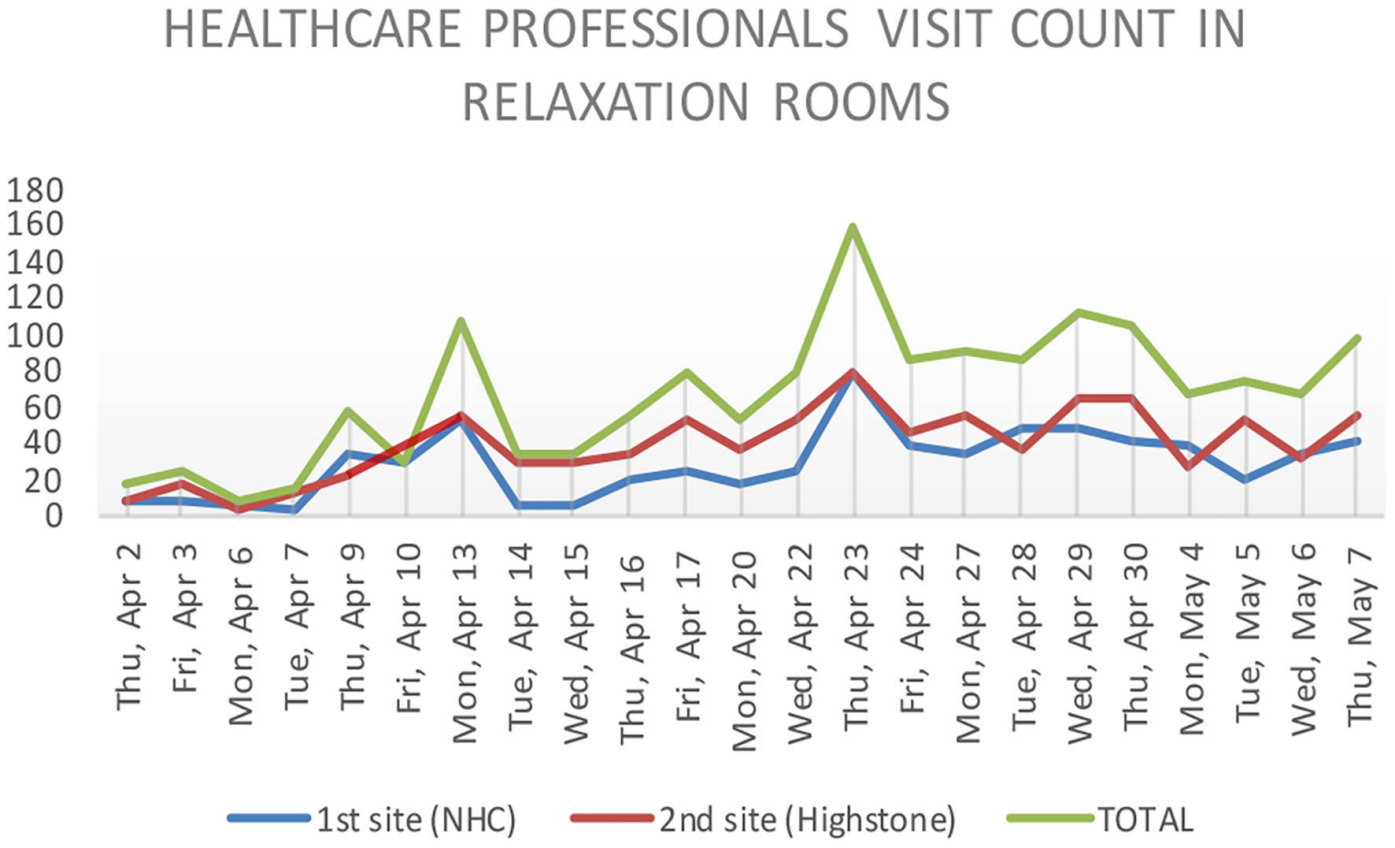
Healthcare professionals' visits to relaxation rooms. Blue and red lines represent the relaxation rooms opened in the two main sites of our hospital. The green line represents the total visit count.

Until May 7, 2020, overall attendance was 2,120 visits for 13,000 agents, with 233 psychological interviews carried out (see Figure 5). The relaxation room has now become a benchmark as a resource place for caregivers. Children's drawings for teams or gifts from local traders dropped off in relaxation rooms also had a positive and supporting impact on healthcare professionals (see Figure 4). However, many caregivers did not allow themselves to visit relaxation rooms or know about their existence.

#### Proactive Mobile Teams

Therefore, CoviPsyHUS created mobile mental health care support teams. Their existence was vital as they enabled us to reach healthcare professionals who could not come to relaxation rooms. Indeed, due to heavy workloads, acute stress-related stupor, or the feeling of being illegitimate in taking care of themselves, many healthcare professionals would not allow themselves a pause. Indeed, stressed workers may shut themselves in and would not seek help, so it is up to mental health teams to be proactive and meet them. The *in-situ* contact in their units offered healthcare workers the opportunity to overcome psychological mechanisms secondary to stress.

In total, 12 proactive mobile teams reached about 1,200 healthcare professionals. We deployed these teams according to a precise map indexing priority units to visit. These mobile teams were composed of caregiver/psychiatrist pairs, thus facilitating interprofessional relationships. Each pair was in charge of a department, enabling a continuous, high-quality connection with specific teams.

Mobile teams distributed psychoeducational documents (including information about sleep disorders, stress management techniques, and advice on talking about the situation with children). Psychoeducation counseling has demonstrated improved stress management with decreased stress levels after the intervention and retrieval of a sense of control over their mental health (20). Moreover, the mobile teams made it possible to reach out through the feeling of isolation, often encountered in highly stressful situations (healthcare workers on intense schedules preventing inter-team exchanges). Re-bonding after a critical experience is essential and recommended (20). Moreover, “Reaching out to caregivers” was seen as a gesture of recognition and taking into account their psychological needs.

Finally, mobile teams units proposed group and individual debriefings to talk about each individual's collective experience. This action was all the most useful for the newly created teams. Indeed, these teams were supposed to split after the crisis period. Individuals would meet their former colleagues, whose exposure to the COVID-19 was different, warranting the implementation of a congruent debriefing. Secondly, Balint groups were proposed (21).

We note that the set-up of mobile teams and a CoviPsyHUS group on social networks led to a significant increase in the use of relaxation rooms. Both these means fostered social connections between healthcare professionals, revealing the importance of substantial and non-stigmatizing mental health information dissemination during a crisis when stress mechanisms are active.

## Discussion and Perspectives

Our first clinical observations (feeling of fear, acute stress, sleep disorders, anxiety, sense of rejection) are consistent with the results of previous studies on epidemics such as SARS (3–6) and with the first Chinese publications for COVID-19 (13–15).

Previous studies (8–10) confirmed the need to systematically integrate mental health support teams dedicated to healthcare professionals in exceptional sanitary situations' care plan to spot and prevent the effects of stress. Following international recommendations (8, 9), our system aimed to act immediately to avoid the progression toward psychiatric complications. Women and frontline health workers fighting against COVID-19 have mostly used CoviPsyHUS system (22). Lai et al. work shown that women and nurses are at high risk of developing mental health problems after exposure to COVID-19 (22). Thus, it made sense that this population noteworthily spent time in the different CoviPsyHUS components.

People under high stress levels could exhibit psychological stupor mechanisms. Therefore, among the critical points to integrate into a mental health care system designed to screen and treat highly exposed professionals (22, 23), we identified the actions promoting contact between healthcare professionals and a mental health system. For this purpose, it is essential to provide extensive and non-stigmatizing dissemination of mental health support information with multimodal communication and have a clear identification of the mental health support system with information on how to access it (3, 23). However, to be even more proactive in these highly stressful situations associated with a heavy workload, we innovated, created proactive mobile teams to identify healthcare professionals in trouble and map the hospital's psychological needs. Early detection by mobile teams could explain a different use of “relaxation rooms” compared to Chinese colleagues, who used them for rest (15). In our hospital, few healthcare professionals came to rest in our relaxation rooms. Stress mechanisms activation may explain this observation (17, 18), but also the benefit of our pro-active and rapid response and the stress management actions implemented. Indeed, “relaxation rooms” helped reducing stress levels and address essential needs. Mental health teams provided stress management techniques during previous epidemic setting, while to our knowledge, they did not provide group workshops (3, 23).

However, in an epidemic like COVID-19, exposure is collective. Respecting the protective measures, we, therefore, proposed group stress management workshops. We observed the positive effect of group learning. Firstly, it generated collective support. Secondly, it was easier for participants to apply the techniques they learned.

Precise health information and concrete infection management measures (social distancing, sufficient protective equipment, sufficient personnel) (3, 15) are associated with lower stress levels and less psychological impact [less anxiety and depression (13)] both for caregivers exposed to SARS (3, 5, 23) and for caregivers exposed to COVID-19 (15, 23) as well as for the general population (13). Like other psychiatric teams in other viral exposition such as SARS (3, 5) we disseminated this medical information to caregivers. However, we also integrated mental health information (23) (psychoeducation), considering different stress levels (COVID-19-related stress, work-related and social stress, personal stress). Moreover, implementing measures to relieve teams under pressure (e.g., relaying communication with families) was an additional element in reducing caregivers' incapacity feelings.

As for other psychological support systems, the mental health support hotline allowed to identify psychological troubles and disseminate stress management advices (3, 14–16). However, these phone consultations widely implemented during the COVID-19 in other hospitals have limitations. There is a lack of medical history data, psychometric psychiatric data, body data, and effective follow-up feedback (3, 14–16). The advantage of CoviPsyHUS compared to other mental healthcare support hotlines is the possibility of an immediate switch to face-to-face individual consultations or to benefit from one of the three other components of the system.

Different resilience and stress management systems emerged during this crisis, but they did not always fit collective and intra-hospital contexts (23). The strength of CoviPsyHUS is to constitute a modular system articulating complementary sub-parts that can be quickly deployed and redeployed when necessary. Its deployment may consider psychological needs and specific constraints (e.g., healthcare professionals agenda, anonymity, distance if work stoppage) at the appropriate time. The system offered various interventions, from early responses to immediate needs (body-centered techniques) to the possibility of complex elaboration of lived experiences (individual psychotherapy and debriefing groups). Moreover, CoviPsyHUS deployed step by step, integrating healthcare professionals' needs from the 1st day of the health crisis. For professionals enduring continuous stress, the system's existence facilitated stress level reduction and had a strong symbolic impact.

Lastly, this system associated the access to both individual (personal dimension) and collective (professional dimension) care within the same entity for healthcare workers' who are subject to double-sided (individual and collective) stress exposure. Indeed, in these situations, mental health care must integrate counseling for the individual and group workshops. It is also a way to recognize the essential involvement of caregivers. Also, work organization arrangements will improve mental health. Finally, this precise adjustment to healthcare professionals' needs seems to optimize health costs. Future studies might clarify the cost-benefit ratio of such interventions.

Though CoviPsyHUS professionals met numerous healthcare professionals in different settings, a limitation is that we did not precisely measure healthcare professionals' disorders (e.g., using surveys or scales). Nonetheless, we also created an online cognitive-behavioral therapy program that will be evaluated in a randomized controlled trial and offer a longitudinal assessment of healthcare professionals' mental health (24). Moreover, many teams conducted surveys worldwide to assess healthcare professionals' mental health during the COVID-19 pandemic (25–27).

Overall, CoviPsyHUS constitutes an innovative, reactive, and transposable mental health prevention and care system. This universal and modular device could serve as a model to deploy in other health (e.g., pandemics) or extra-health crises (e.g., nuclear or chemical risks situations), causing prolonged stress.

## Data Availability Statement

The original contributions presented in the study are included in the article/supplementary material, further inquiries can be directed to the corresponding author/s.

## Ethics Statement

Ethical review and approval was not required for the study on human participants in accordance with the local legislation and institutional requirements. Written informed consent for participation was not required for this study in accordance with the national legislation and the institutional requirements. Written informed consent was obtained from the individual(s) for the publication of any potentially identifiable images or data included in this article.

## Author Contributions

JR: CoviPsyHus project co-responsible, mobile team supervisor, and sleep psychoeducation workshops manager. AM: CoviPsyHus project supervisor. CP: relaxation rooms supervisor. DM: CoviPsy67 supervisor. MF: doctor involved in CoviPsyHus and CoviPsy67. AG: doctor responsible for the part of the system dedicated to the families. J-JVH: doctor in charge of a covid unit who was a privileged contact for the feedback of clinical experience CS: local reviewer and sleep psychoeducation workshops supervisor. PV: CoviPsy project supervisor.

## Conflict of Interest

The authors declare that the research was conducted in the absence of any commercial or financial relationships that could be construed as a potential conflict of interest.
